# Comparison of diagnostic performance between conventional and ultrasensitive rapid diagnostic tests for diagnosis of malaria: A systematic review and meta-analysis

**DOI:** 10.1371/journal.pone.0263770

**Published:** 2022-02-10

**Authors:** Yonas Yimam, Mehdi Mohebali, Mohammad Javad Abbaszadeh Afshar

**Affiliations:** 1 Department of Medical Parasitology and Mycology, School of Public Health, Tehran University of Medical Sciences, Tehran, Iran; 2 Department of Biology, Faculty of Natural and Computational Sciences, Woldia University, Woldia, Ethiopia; 3 Centers for Research of Endemic Parasites of Iran (CREPI), Tehran University of Medical Sciences, Tehran, Iran; 4 Department of Medical Parasitology and Mycology, School of Medicine, Jiroft University of Medical Sciences, Jiroft, Iran; Quensland University of Technology, AUSTRALIA

## Abstract

**Background:**

Successful malaria treatment, control and elimination programs require accurate, affordable, and field-deployable diagnostic tests. A number of studies have directly compared diagnostic performance between the new ultrasensitive rapid diagnostic test (us-RDT) and conventional rapid diagnostic test (co-RDT) for detecting malaria. Thus, we undertook this review to directly compare pooled diagnostic performance of us-RDT and co-RDT for detection of malaria.

**Methods:**

PubMed, Web of Science, Scopus, Embase, and ProQuest were searched from their inception until 31 January 2021 accompanied by forward and backward citations tracking. Two authors independently assessed the quality of included studies by RevMan5 software (using the QUADAS-2 checklist). Diagnostic accuracy estimates (sensitivity and specificity and others) were pooled using a random-effect model and 95% confidence interval (CI) in Stata 15 software.

**Results:**

Fifteen studies with a total of 20,236 paired co-RDT and us-RDT tests were included in the meta-analysis. Molecular methods (15 studies) and immunoassay test (one study) were used as standard methods for comparison with co-RDT and us-RDT tests. The pooled sensitivity for co-RDT and us-RDT were 42% (95%CI: 25–62%) and 61% (95%CI: 47–73%), respectively, with specificity of 99% (95%CI: 98–100%) for co-RDT, and 99% (95%CI: 96–99%) for us-RDT. In asymptomatic individuals, the pooled sensitivity and specificity of co-RDT were 27% (95%CI: 8–58%) and 100% (95%CI: 97–100%), respectively, while us-RDT had a sensitivity of 50% (95%CI: 33–68%) and specificity of 98% (95%CI: 94–100%). In low transmission settings, pooled sensitivity for co-RDT was 36% (95%CI: 9 76%) and 62% (95%CI: 44 77%) for us RDT, while in high transmission areas, pooled sensitivity for co RDT and us RDT were 62% (95%CI: 39 80%) and 75% (95%CI: 57–87%), respectively.

**Conclusion:**

The us-RDT test showed better performance than co-RDT test, and this characteristic is more evident in asymptomatic individuals and low transmission areas; nonetheless, additional studies integrating a range of climate, geography, and demographics are needed to reliably understand the potential of the us-RDT.

## Introduction

Alongside the scale-up of malaria prevention and treatment interventions, tremendous progress has made in reducing global malaria cases incidence and malaria deaths, such that between 2000 and 2015, malaria incidence rates fell 41% globally, malaria mortality rates were reduced by 62% and 17 countries eliminated malaria [1]. Bolstered by these remarkable gains, Global Technical Strategy for malaria (2016–2030) develops goals to eliminate malaria by 2030 from at least 35 countries in which malaria was transmitted in 2015 and reduce global malaria incidence by 90% compared to 2015 [2]. In this context, active malaria case detection are a key component and this requires diagnostic tools capable of detecting low parasitemic infections in low endemicity regions and asymptomatic infections in high transmission settings [3].

Microscopic examination of peripheral blood smear for the presence of *Plasmodium* parasite remains the mainstay of malaria diagnosis; nonetheless, it has low sensitivity for detecting low- density parasitemia [4–6]. Also, microscopy needs trained personnel and sufficient laboratory reagents and equipment [7]. While nucleic acid amplification-based diagnostic tests can diagnose low-density infection, they are impractical for use in resource-constrained settings due to the need for sophisticated laboratory facilities, high-cost and highly trained staff [8]. Conventional malaria rapid diagnostic tests (co-RDTs) are easy to use, low-cost, rapid, and field deployable tool for the detection of malaria antigens, but co-RDTs have limitation for the detection of low density and asymptomatic malaria infections [9]. For successful malaria control and elimination, routine surveillance of low-density infections is crucial and this requires highly sensitive and field- deployable tests [3].

New malaria detection test, such as Alere™ Ultra-sensitive Malaria Ag *P*. *falciparum* RDT (us-RDT) test, has been recently developed for the detection of low malaria parasite density [10]. This us-RDT test, similar to many co-RDT tests, is designed in an immunochromatographic strip platform to detect histidine-rich protein 2(HRP2), but with improved analytical sensitivity (i.e. a detection threshold lower than co-RDTs) [11]. Various studies have assessed diagnostic performance of us-RDT and co-RDT for the detection of malaria in the same study population [12–16]. These studies demonstrated a wild discrepancy in the magnitude of us-RDT performance compared to co-RDT; considerable studies highlighted us-RDT outperform co-RDT with different magnitudes [12, 13, 15, 17], whereas one single study found no difference in sensitivity of us-RDT compared to co-RDT [16]. A systematic review and meta-analysis was carried out recently [18]; however, this meta-analysis had missed studies [10, 12, 19, 20] and did not evaluate diagnostic accuracy of us-RDT and co-RDT in relation to malaria transmission settings. Therefore, this review was conducted to establish summary estimates of us-RDT diagnostic performance compared to co-RDT, taking into account missed studies and malaria transmission settings.

## Methods

This systematic review and meta-analysis was conducted according to Preferred Reporting Items for Systematic Reviews and Meta-analyses of Diagnostic Test Accuracy studies (PRISMA-DTA) guidelines and Cochrane Handbook for Systematic Reviews of Diagnostic Test Accuracy [21]. PRISMA Checklist is presented in Supplementary information (**S1 Checklist**).

### Data sources and searches

We systematically searched the following databases; PubMed, Web of Science, Scopus, Embase, and ProQuest, from the earliest available dates of indexing through 31 January 2021. The searches were restricted to the English language. The search strategies were based on all possible combinations of the following Medical Subject Headings (MeSH) terms and keywords; ’’Infection, Plasmodium ’’, ’’Plasmodium Infections’’, ’’malaria’’, ’’ultra-sensitive’’, ’’ultrasensitive’’, ’’highly-sensitive’’, ’’ highly sensitive’’, ’’high-sensitive’’, ’’high sensitive’’, ’’diagnosis’’, ’’ rapid diagnostic test’’, ’’detection ’’, ’’rapid diagnosis’’, and ’’rapid test ’’. Additionally, we manually secerned reference lists of eligible studies and relevant reviews and forward citations tracking using Google Scholars. When there were incomplete data for the 2x2 table, we contacted the corresponding authors through email. The description of the exact search is available in **S1 File.**

### Eligibility criteria and studies selection

In this study, we included studies that fulfill the following criteria; i) original studies that directly compare diagnostic test accuracy us-RDT and co-RDT in the same population, (ii) studies directly compare diagnostic test accuracy of us-RDT and co-RDT with reference standard tests, (iii) studies that contain data for 2X2 table completion (True Positive (TP), False Positive (FP), False Negative (FN), True Negative (TN)). The following types of studies were excluded; i) studies reported diagnostic accuracy of only us-RDT or co-RDT, but not both, ii) relevant full-text studies with inadequate data for 2X2 tables after two email contact of corresponding authors, iii) non-original studies, such as reviews, conference papers, and letters.

Records obtained using literature searches were kept in EndNote X 8 and the selection of eligible studies was conducted independently by two authors (YY and MJA) based on pre-determined eligibility criteria. First, we removed duplicate studies and then we reviewed titles and abstracts of the remaining studies. Secondly, titles and abstracts not pertinent to the objectives of this study were removed. Following the removal of duplicate records and titles and abstracts screening, full-text of the remaining studies were thoroughly reviewed and ineligible studies were further removed. Fourthly, data were extracted from full-text studies that met eligible criteria. Disagreements between two authors were resolved through discussion.

### Data extraction and quality assessment

Data extraction format was prepared to compile data from eligible studies. For each study, we extracted information such as study author/s and year, country where the study conducted, level of malaria endemicity, (low or/and medium or/and high transmission settings), study population(asymptomatic or/and symptomatic), reference and index tests, and complete data for 2X2 table (TP, TN, FP, FN). Each country classified a territory or geographic area based on confirmed malaria cases in a particular year, characterized as low, medium, or high malaria intensity transmission per 1,000 people under surveillance [22]. Consequently, we recorded low, medium, or high malaria transmission based on the reported stratification/classification of malaria endemicity in the included studies. When the data were insufficient to complete the 2X2 table, corresponding authors were contacted through email to acquire masked data. Data extraction was carried out independently by two authors (YY and MJA) and the discrepancy was resolved by consensus.

### Quality assessment

For quality assessment, we used the revised tool for the Quality Assessment of Diagnostic Accuracy Studies (QUADAS‐2) [23]. The QUADAS‐2 tool includes four key domains; (1) patient selection, (2) index test, (3) reference standard, and (4) flow and timing. Review Manager 5(RevMan 5.4.1) was used to generate the risk of bias summary and graph. Two authors (YY and MJA) independently assessed the quality of included studies using the QUADAS‐2 tool and any disagreement was resolved through consensus.

### Data analysis

We constructed 2X2 contingency tables consisting of TP, TN, FP, and FN. We calculated pooled diagnostic measures, including sensitivity, specificity, positive likelihood ratio, negative likelihood ratio, and diagnostic odds ratio by STATA version 15 software using *midas* commands. Between studies heterogeneity was examined using Cochrane’s Q test and quantified with the inconsistency (I^2^) test, using a random-effects model. Summary receiver operating characteristic (SROC) was drawn and the area under the summary receiver operating characteristic curve (AUC) was calculated to estimate overall test performance. We also performed subgroup analysis based on the level of malaria endemicity. Publication bias was evaluated using Deeks funnel plot asymmetry test [24].

## Results

### Search results and basic characteristics of included studies

**Fig 1:**
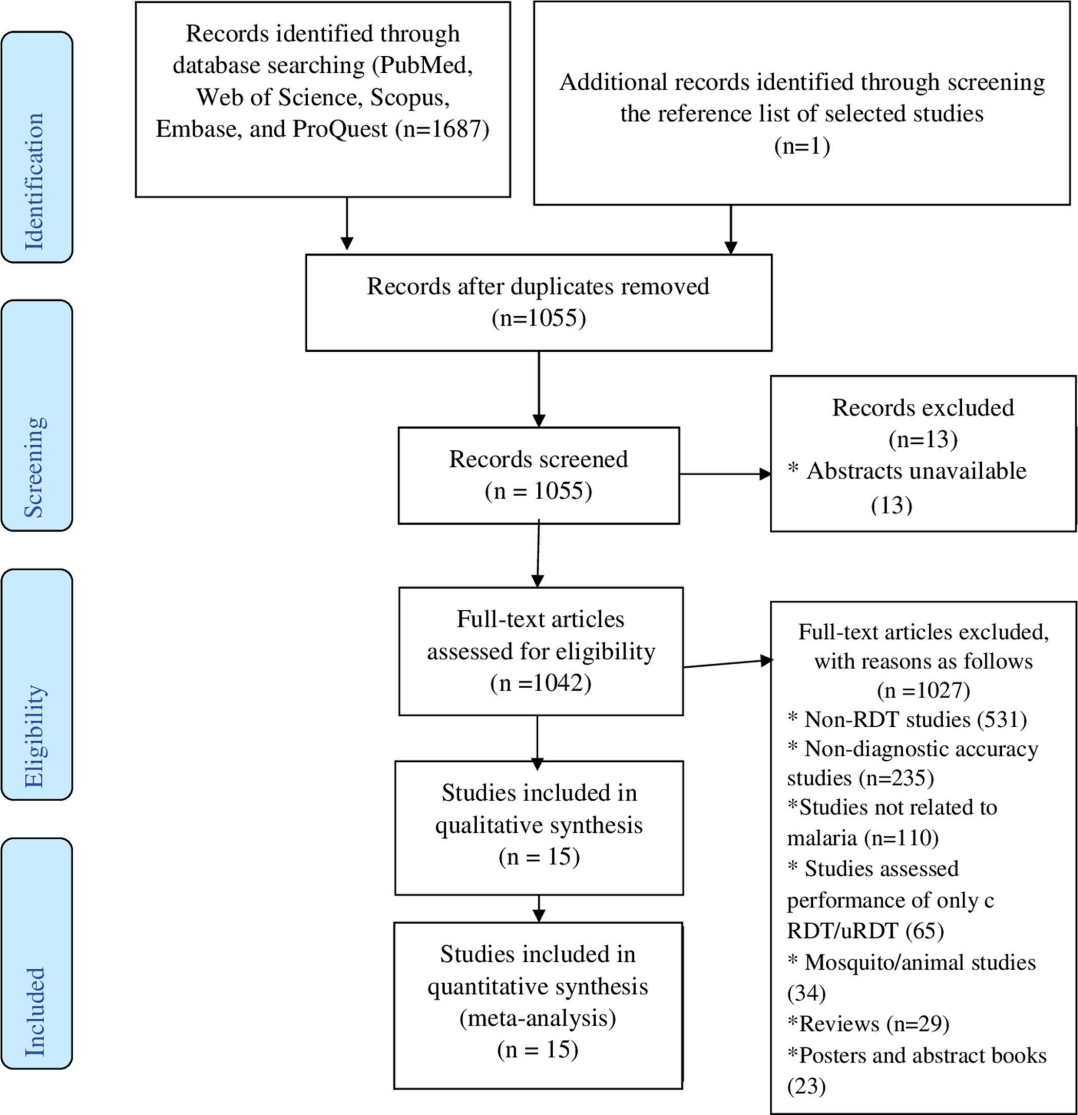
Flow diagram showing literature search and study selection process used to capture studies that compared diagnostic performance of co-RDT and us-RDT in the same populations, 2021.

A total of 1687 records were retrieved from database searches, of which 633 were duplicate records. After the exclusion of duplicate records, the remaining records (1055) were screened for titles and abstracts and 13 of these were removed due to the unavailability of their abstracts. 1042 studies were left for full-text evaluation, and 1027 of these were removed with reasons. The most prominent reasons for exclusion are as follow: no report of RDT in studies (531), studies not evaluated diagnostic test accuracy (235), studies are not related to malaria (110), and studies assessed the performance of only co-RDT or only us-RDT rather than both tests (65) (**Fig 1**). As a result, 15 studies were selected for the present study. Additional searches of reference lists of selected studies and relevant reviews resulted in the inclusion of one additional study [25]. Overall, a total of 16 studies (conducted in 17 malaria transmission settings) were included in the final quantitative synthesis.

The basic characteristics of the included studies are presented in **S1 Table**. All included studies were conducted from 2017 to 2021. In total, 20,236 paired co-RDT and us-RDT tests were compared with standard methods. Seven, five, and two included studies, respectively, were based on asymptomatic populations only, both symptomatic and asymptomatic populations, and symptomatic populations only, whereas two studies did not report the clinical status of the study populations. All included studies are from 11 countries: Myanmar (3 studies) [10, 25, 26], Tanzania (2 studies) [14, 27], Colombia (2 studies) [12, 28], Uganda (2 studies) [10, 19] and one each in Mozambique [29], Haiti [30], Indonesia [31], Cambodia [16], Ethiopia [15], Benin [32], and Papua New Guinea [13]. Seven studies were carried out in each of high and low endemicity region, two studies in medium endemicity region and one in moderate and high endemicity. All included studies used the same brand of us-RDT (Alere™ Malaria Ag P.f Ultra-Sensitive rapid diagnostic test). Standard methods used for comparison with conventional and ultrasensitive tests were quantitative Polymerase Chain Reaction (PCR) (9 studies), nested PCR (6 studies), and HRP2 bead-based immunoassay (one study). Gene targets for molecular methods were 18s ribosomal RNA, *var* gene sequences, 18s ribosomal DNA and genomic DNA in, eight, two, one, and one studies, respectively, while gene targets for molecular methods were not reported in four included studies.

### Methodological quality of included studies

**Fig 2:**
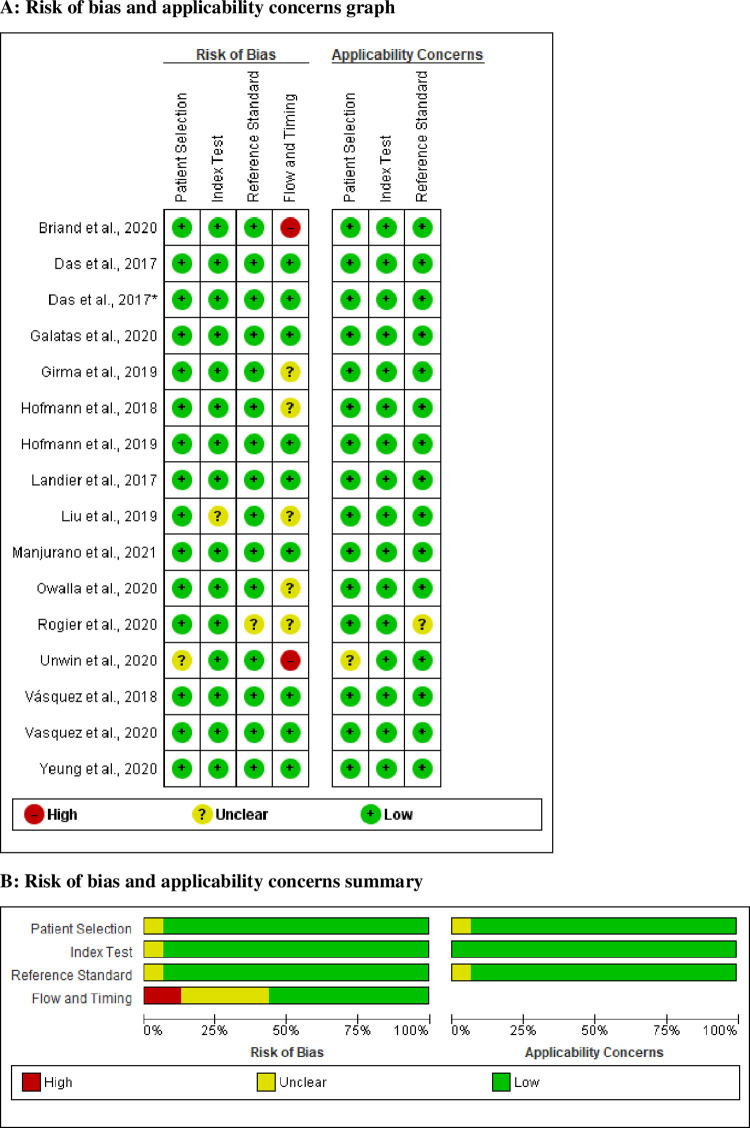
A. Risk of bias and applicability concerns graph of included studies that compared diagnostic performance of co-RDT and us-RDT in the same populations, between 2017 and 2021. B. Risk of bias and applicability concerns summary of included studies that compared diagnostic performance of co-RDT and us-RDT in the same populations, from 2017 to 2021.

As pointed out in the risk of bias and applicability concerns graph (**Fig 2A**) and summary (**Fig 2B**), the overall quality of included studies was adequate. Low risk of bias for participants’ selection was observed in 93.75% of included studies, while the reaming 6.25% of studies showed an unclear risk of bias. The quality of confirmation of reference methods and the use of index testes were considered sufficient in 97% of studies. None of the included is case-control. Seven out of fifteen included studies did not provide information about the selection of study participants.

### Meta-analysis

#### Diagnostic accuracy of co-RDT and us-RDT for the detection of malaria

**Fig 3:**
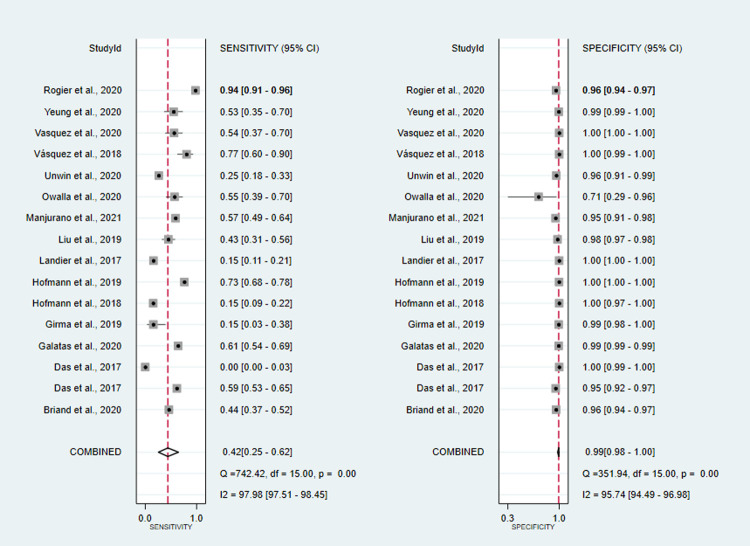
Forest plot of sensitivity and specificity of the co-RDT test between 2017 and 2021.

**Fig 4:**
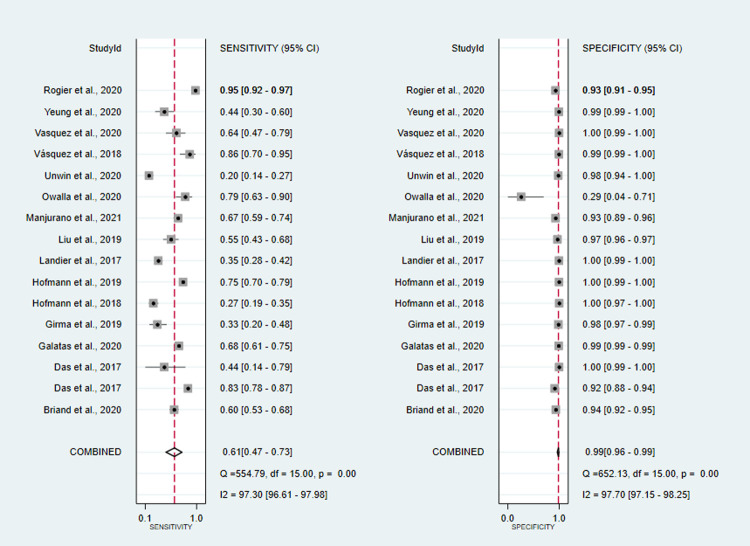
Forest plot of sensitivity and specificity of us-RDT test between 2017 and 2021.

co-RDT sensitivity ranged from 15% (95%CI: 3–38%) to 94% (95%CI: 91–96%), while us-RDT sensitivity ranged from 20% (95%CI: 14–27%) to 95% (95%CI: 92–97%), with specificity ranged from 71% (95%CI: 29–96%) to 100% (95%CI: 99.9–100%) and from 29% (95%CI: 4–71%) to 100% (95%CI: 99–100%) for co-RDT and us-RDT, respectively. The pooled sensitivity were 42% (95%CI: 25–62%) for co-RDT, 61% (95%CI: 47–73%) for us-RDT and specificity were 99% (95%CI: 98–100%) for co-RDT, 99% (95%CI: 96–99%) for us-RDT (**Figs 3** and **4**). The pooled estimates for the positive likelihood ratio (PLR), negative likelihood ratio(NLR), and diagnostic odds ratio(DOR) for co-RDT were 61.8 (95%CI: 21.5–177.5), 0.58 (95%CI: 0.41–0.82) and 106 (95%CI: 36–328), respectively, and the overall PLR, NLR, and DOR for us-RDT were 42.3 (95%CI: 17.9–99.7), 0.40 (95%CI: 0.28–0.55) and 107 (95%CI: 45–253), respectively. The AUC for co-RDT and us-RDT were 0.94 (95%CI: 0.92–0.96) and 0.93 (95%CI: 0.90–0.95), respectively.

**Fig 5:**
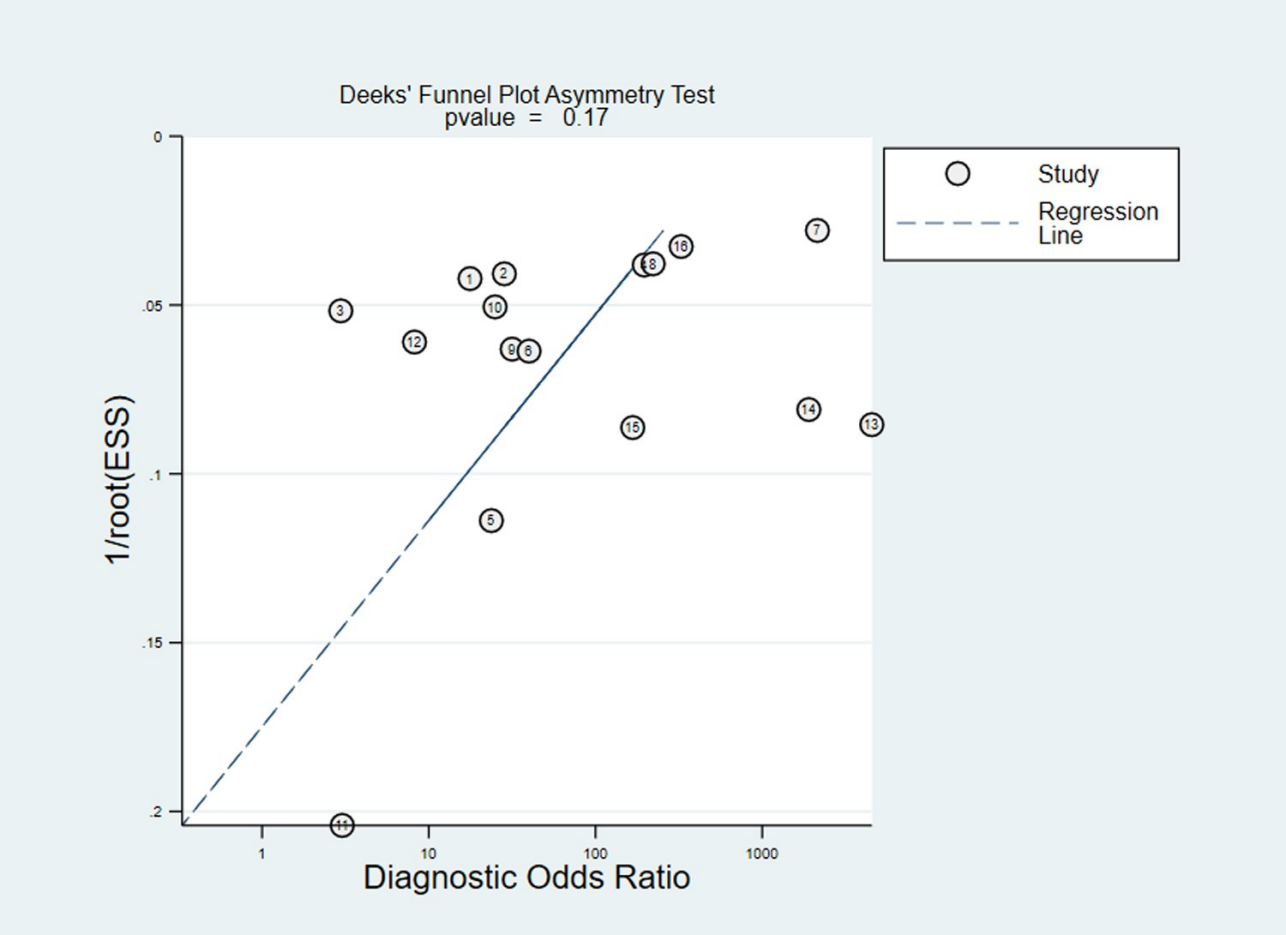
Deeks’ funnel plot asymmetry test for publication bias of co-RDT test, 2017 to 2021. ESS; effective sample size. P value <0.005 were considered significant.

**Fig 6:**
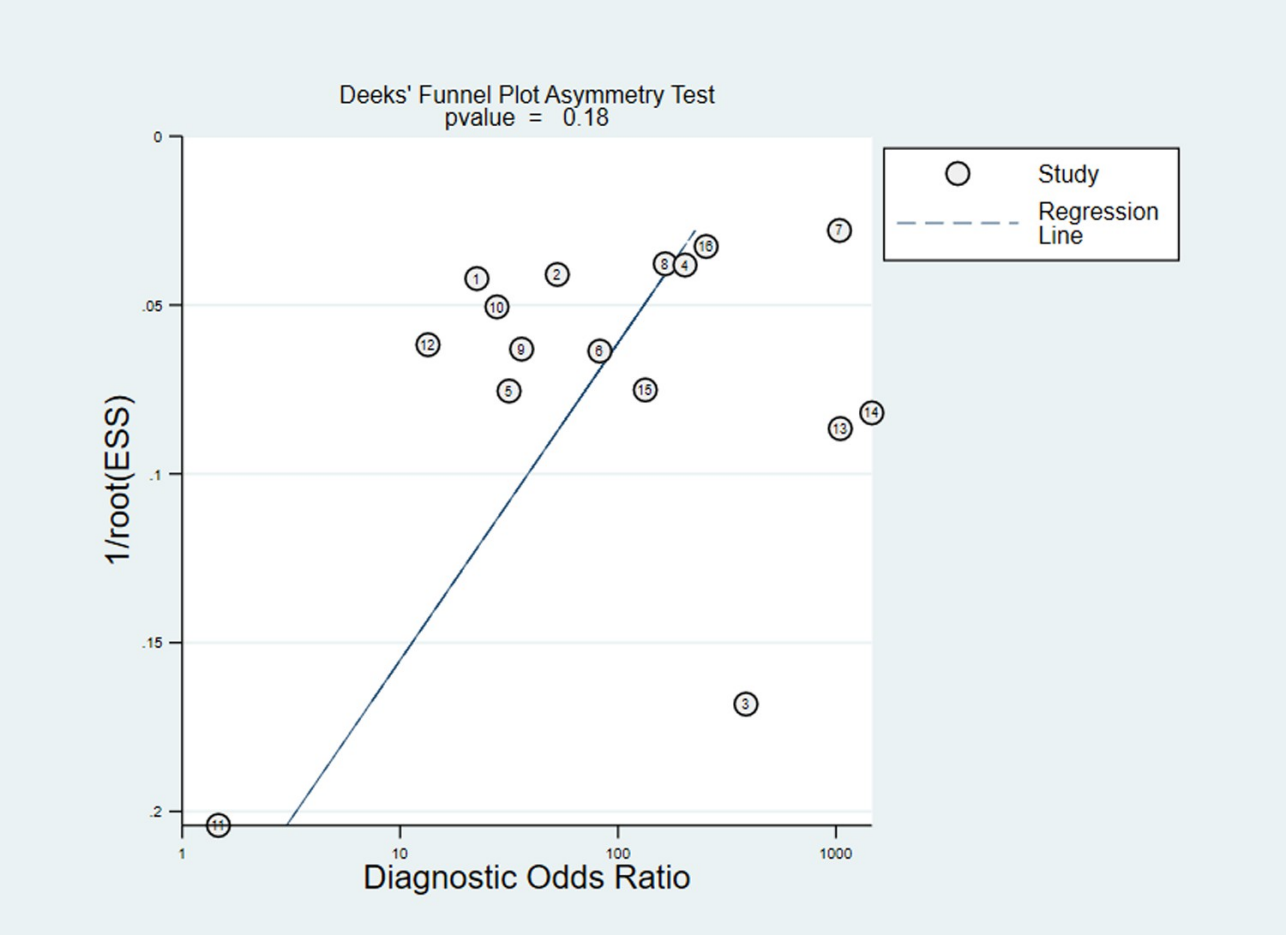
Deeks’ funnel plot asymmetry test for publication bias of us-RDT test, 2017 to 2021. ESS; effective sample size. P value <0.005 were considered significant.

High heterogeneity of sensitivity and specificity were observed in co-RDT (Q = 742.42, I^2^ = 97.98, P<0.001 and Q = 351.94, I^2^ = 95.71, P<0.001, respectively) and us-RDT (Q = 554.79, I^2^ = 97.3, P<0.001 sensitivity and Q = 652.13, I^2^ = 97.70, P<0.001 for specificity, respectively) tests. Sub-group analysis were carried out based on reference tests used and level of malaria endemicity. In low transmission settings, pooled sensitivity for co-RDT was 36% (95%CI: 9–76%) and 62% (95%CI: 44–77%) for us-RDT, while in high transmission areas, pooled sensitivity for co-RDT and us-RDT were 62% (95%CI: 39–80%) and 75% (95%CI: 57–87%), respectively (**Table 1**). When nested-PCR and quantitative-PCR used as reference tests, the sensitivity and specificity of co-RDT were 51% (95%CI: 10.37–65%) and 99% (95%CI: 96–100%) and 28% (95%CI: 11–57%) and 100% (95%CI: 97–100%), respectively. The sensitivity and specificity of us-RDT when nested-PCR and quantitative-PCR used as a reference tests was 56% (95%CI: 37–74%) and 99% (95%CI: 97–100%) and 58% (95%CI: 42–72%) and 99% (95%CI: 94–100%), respectively. The Deeks’ funnel plot asymmetry test of DOR did not show substantial asymmetry (P = 0.17 for co-RDT and P = 0.18 for us-RDT), pinpointing that there could be no detectable publication bias (**Figs 5** and **6**).

**Table 1:** Sub-group analysis of included studies that compared diagnostic performance of co-RDT and us-RDT in the same populations, between 2017 and 2021, based on diagnostic methods, transmission settings and symptomatic malaria patients.

Test	Category	No. studies	TP	FP	FN	TN	Sensitivity (95% CI)	Specificity (95% CI)	Positive likelihood ratio (95% CI)	Negative likelihood ratio (95% CI)	Diagnostic odds ratio (95% CI)
Co-RDT	Reference method										
	Nested-PCR	6	229	79	262	6412	0.51(0.37–0.65)	0.99(0.96–1.00)	86.8(10.6–707.6)	0.49(0.36–0.66)	178(18–1790)
	qPCR	9	687	94	822	10650	0.28(0.11–0.57)	1.00(0.97–1.00)	59.5(12.8–276.9)	0.72(0.51–1.01)	83(18–384)
	Transmission settings										
	Low transmission	7	169	65	383	7932	0.36(0.09–0.76)	1(0.99–1.0)	281.9(38.5–2063.3)	0.65(0.35–1.20)	437(46–4109)
	High transmission	7	943	120	426	9284	0.62(0.39–0.80)	0.98(0.93–0.99)	29.4(9.0–95.5)	0.39(0.22–0.70)	75(19–302)
Us-RDT	Reference method										
	qPCR	9	871	149	538	10695	0.58(0.42–0.72)	0.99(0.94–1.00)	42.3(10.2–176)	0.43(0.30–0.61)	99(27–368)
	Nestd-PCR	6	259	102	258	6363	0.56(0.37–0.74)	0.99(0.97–1.00)	49.3(15.6–155.3)	0.44(0.29–0.69)	111(28–449)
	Transmission settings										
	Low transmission	7	231	95	217	8006	0.62(0.44–0.77)	0.99(0.99–1.00)	99.2(43.4–226.4)	0.38(0.25–0.60)	258(99–673)
	High transmission	7	1070	182	314	8757	0.75(0.57–0.87)	0.94(0.79–0.98)	12.6(3.4–47.4)	0.27(0.15–0.48)	48(10–226)
Co-RDT	Symptomatic malaria patients	3	378	15	173	3407	0.69(0.65–0.72)	0.99(0.99–1.00)	156.51(94.16–260.13)	0.32(0.28–0.36)	496.28(289.75–850.04)
US-RDT	Symptomatic malaria patients	3	402	24	149	3398	0.73(0.69–0.76)	0.99(0.99–1.00)	104.03(69.60–155.49)	0.27(0.24–0.31)	381.99(245.20–595.09)

Abbreviations: TP, true positive; FP, false positive; FN, false negative; TN, true negative; q, quantitative; PCR, polymerase chain reaction.

#### Diagnostic accuracy of co-RDT and us-RDT for the detection of asymptomatic and symptomatic malaria

**Fig 7:**
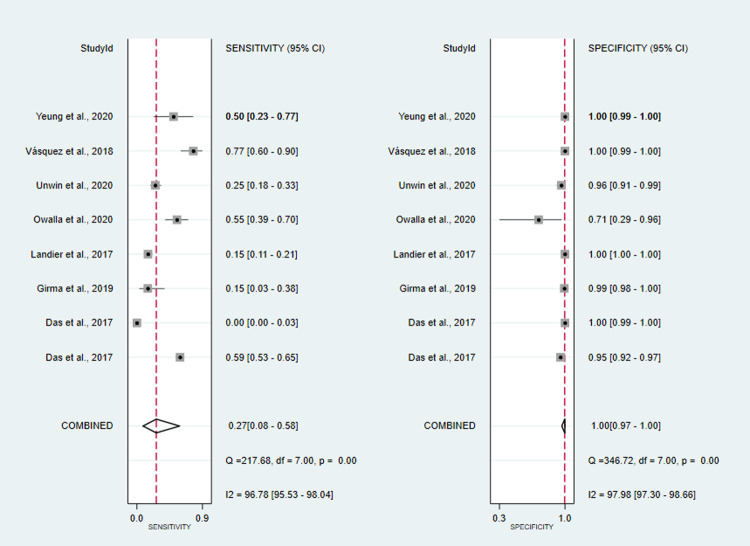
Forest plot of sensitivity and specificity of the co-RDT test in asymptomatic individuals, from 2017 to 2021.

**Fig 8:**
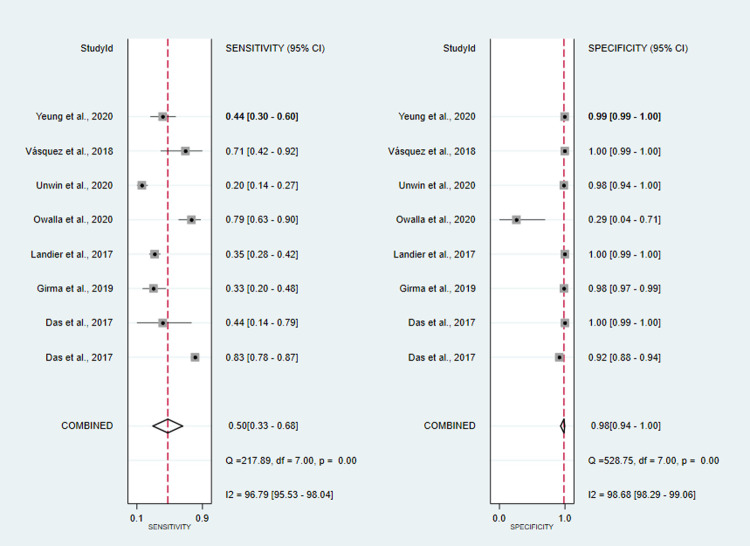
Forest plot of sensitivity and specificity of us-RDT test in asymptomatic individuals, from 2017 to 2021.

A total of eight studies assessed the diagnostic accuracy of co-RDT and us-RDT in the same asymptomatic study population. The pooled sensitivity and specificity of co-RDT was 27% (95%CI: 8–58%) and 100% (95%CI: 97–100%) (**Fig 7**), respectively, while us-RDT had a sensitivity 98% (95%CI: 94–100%) and specificity of 50% (95%CI: 33–68%) (**Fig 8**), respectively. The AUC was 90% (95%CI: 87–92%) in co-RDT test and 86% (95%CI: 83–89%) in us-RDT test. In symptomatic populations, co-RDT sensitivity and specificity was 69% (95%CI: 65–72%) and 99% (95%CI: 99–100%), respectively, while us-RDT sensitivity and specificity was 73% (95%CI: 69–76%) and 99% (95%CI: 99–100%), respectively (**Table 1**).

## Discussion

As many countries progress from malaria control to elimination, ultrasensitive and field-deployable diagnostic tests that are capable of detecting both symptomatic and asymptomatic plasmodium infections are of paramount importance to guide and measure the success of elimination strategies [3, 33]. In light of this, efforts have been made to develop highly sensitive diagnostic tests, including recently developed highly sensitive RDT (Alere™ Malaria Ag Pf ultra-sensitive RDT) [34]. Various studies have assessed diagnostic accuracy of the newly developed highly sensitive RDT and conventional RDT testes. This review, therefore, was undertaken to estimate pooled diagnostic test accuracy of co-RDT and us-RDT in the same study populations. In this review, us-RDT (61% (95%CI: 47-73%) showed better pooled sensitivity than co-RDT (42% (95%CI: 25-62%)), while similar pooled specificity were observed between co-RDT and us-RDT (99% (95%CI: 98–100%) for co-RDT vs. 99% (95%CI: 96–99%) for us-RDT). It was found that the pooled sensitivity of us-RDT test was better than co-RDT in low malaria transmission settings (62% (95%CI: 44–77%) for us-RDT vs. 36% (95%CI: 9–76%) for co-RDT) and asymptomatic study population (50% (95%CI: 33–68%) for us-RDT vs. 27% (95%CI; 8–58%) for co-RDT).

Our systematic review and meta-analysis revealed improved sensitivity of us-RDT compared to co-RDT; however, high heterogenity in sensitivity across included studies was observed, which could be related to variations in clinical status of study populations, age of study participants, parasite density and endemicity of malaria in study areas. In a study conducted among pregnant women in Benin, the sensitivity of co-RDT(44.2%) and us-RDT(60.5%) were shown to be similar [32]. Primary studies carried out in Ethiopia (7.3% for co-RDT vs.50% for us-RDT) [15], and Papua New Guinea (15% for co-RDT vs. 27% for us-RDT) [13] demonstrated lower sensitivity compared to this meta-analysis. Conversely, the pooled sensitivity of this review was lower than the sensitivity of studies conducted in Indonesia (62% for co-RDT vs. 84% for us-RDT) [31] and Colombia (64.3% for co-RDT vs.71.4%uRDT) [12]. The discrepancy in the sensitivity may be related to the difference in the endemicity of malaria in studies, study population, sample size and reference methods.

Malaria transmission intensity is stratified for a given country, territory, or geographic area based on confirmed malaria cases in a specific year, expressed per 1,000 population under surveillance. According to WHO indicative metrics of malaria transmission intensity, areas of high transmission are characterized by an annual parasite incidence of about 450 or more cases per 1000 population and *P*. *falciparum* prevalence rate of ≥35%, while low transmission have an annual parasite incidence of 100–250 cases per 1000 population and a prevalence of *P*. *falciparum/P*. *vivax* of 1–10% [22] Nonetheless, each country can conduct a stratification to classify geographical units according to local malaria transmission [22]. For detecting low density infection and monitoring malaria transmission in low-transmission areas, more sensitive diagnostic tests are required [36]. Currently available commercial co-RDTs are not capable of detecting malaria in endemic areas with a parasite density of 100,000 parasites/ml, however us-RDT is.

In malaria elimination campaign, asymptomatic individuals harbor *Plasmodium* parasites with no overt clinical signs and low level of parasite density, making difficult to detect using co-RDTs [35]. To bridge this gap, us-RDT test was introduced to be able to able to detect more asymptomatic infection below 200 p/μl parasite density [34]. The pooled sensitivity of us-RDT in asymptomatic individuals in this study was 1.85 fold higher than co-RDT test. The specificities of the two tests were similar (100% for co-RDT and 98% for us-RDT). A study conducted among asymptomatic individuals from Myanmar revealed that us-RDT detected 2- fold more *Plasmodium* infections than co-RDT [25]. A significant proportion of asymptomatic individuals harbor the lowest parasite density, as low as 1000 parasites per ml, which is below the limit of detection threshold of commonly used RDTs(with low a lower detection limit of 100,000 parasites per ml) [36, 37]. As a result, studies using conventional RDTs, which are capable of detecting parasite density exceeding 100,000 parasites/ml may fail to detect some proportions of *Plasmodium* infection, thus the diagnostic performance of co-RDT reduce [34]. The possible superior performance of us-RDT to co-RDT for detecting asymptomatic malaria infection may result from the improved performance of us-RDT, with a capacity to detect 100–10,000 p/ml [34].

This study had some limitations. First, considerable heterogenity among included studies was observed. To explore the existing source of heterogeneity, we performed sub-group analysis based on reference standards and level of malaria endemicity; nevertheless, we failed to find the factor responsible for heterogeneity. Second, all included studies are from a limited number of countries (11 countries). Third, literature searches were performed only in English, which may introduce language bias. Fourth, due to the lack of adequate information, subgroup analysis based on other factors such as age, sex, and parasite density were not explored. Fifth, this review protocol was not registered in PROSPERO since we have initiated literature searches prior to protocol registration.

In conclusion, this review confirmed higher overall performance of us-RDT, compared to co-RDT, to detect *Plasmodium* infections. Considerably higher pooled sensitivity of us-RDT observed particularly for detection of malaria in areas of low transmission settings and asymptomatic individuals, suggesting the superior performance of us-RDT to co-RDT for surveillance of asymptomatic malaria asymptomatic individuals that are key for malaria elimination. Taking into account substantial heterogenity across included studies, further large-scale, well-designed, and multi-center studies including various study participants symptoms, age, sex, parasite density, and malaria transmission settings of the study areas are needed to reliably understand the true performance of us-RDT test for malaria surveillance.

## Supporting information

S1 Table The basic characteristics of the included studies that compared diagnostic performance of co-RDT and us-RDT in the same populations, 2017 to 2021. (DOCX)

S1 Checklist PRISMA diagnostic test accuracy checklist. PRISMA-DTA guideline.
(DOC)

S1 File Search strategies to retrieve as many studies as possible that compared diagnostic performance of co-RDT and us-RDT in the same populations, 2021. (DOCX)
