# Supplementary material for: Comparison of diagnostic performance between conventional and ultrasensitive rapid diagnostic tests for diagnosis of malaria: A systematic review and meta-analysis
Source: PLoS One. 2022 Feb 10;17(2):e0263770. doi: 10.1371/journal.pone.0263770 (PMC8830612; doi:10.1371/journal.pone.0263770)
Supplement: S1 Table — (DOCX) [file pone.0263770.s001.docx]

S1 Table. The basic characteristics of the included studies that compared diagnostic performance of conventional RDT and ultrasensitive RDT (Alere™ Ultra-sensitive Malaria Ag *P. falciparum* RDT) in the same populations, 2017 to 2021

| Author/s | Country | Diagnostic test | | | | | | | | Study design | Study  settings | Age range/s | Total sample size | Conventional  RDT  brand | Reference  Standard  test | Transmission  setting | Clinical status of study  populations |
| --- | --- | --- | --- | --- | --- | --- | --- | --- | --- | --- | --- | --- | --- | --- | --- | --- | --- |
|  |  | Conventional RDT | | | | Ultrasensitive RDT | | | |  |  |  |  |  |  |  |  |
|  |  | TP | FP | FN | TN | TP | FP | FN | TN |  |  |  |  |  |  |  |  |
| Briand et al., 2020 | Benin | 76 | 33 | 96 | 737 | 104 | 49 | 68 | 721 | Ret | Cl | >15 years | 942 | SD Bioline Malaria Ag Pf | 18S rDNA qPCR | High | Asymptomatic&  Symptomatic |
| Das et al., 2017 | Uganda | 163 | 16 | 113 | 315 | 218 | 29 | 45 | 315 | Coh | Co | > 6 months | 607 | SD Bioline Malaria Ag Pf | 18s rRNA qRTPCR | High | Asymptomatic |
| Das et al., 2017 | Myanmar | 0 | 0 | 9 | 484 | 4 | 1 | 5 | 369 | Coh | Co | > 6 months | 493 | SD Bioline Malaria Ag Pf | 18s rRNA qRTPCR | Low | Asymptomatic |
| Galatas et al., 2020 | Mozambique | 110 | 34 | 69 | 4183 | 122 | 44 | 57 | 4173 | Cros | Co | All ages | 4396 | SD Bioline Malaria Ag Pf | RT-qPCR | High | Asymptomatic&  Symptomatic |
| Girma et al., 2019 | Ethiopia | 3 | 4 | 17 | 538 | 16 | 8 | 32 | 506 | Cros | Co | All ages | 562 | CareStart RDT | RT-qPCR | High | Asymptomatic |
| Hofmann et al., 2018 | Papua New Guinea | 20 | 0 | 115 | 112 | 36 | 0 | 99 | 112 | Cros | Co | >5 years | 247 | NR | varATS qPCR | Medium | NR |
| Hofmann et al., 2019 | Tanzania | 261 | 4 | 96 | 3154 | 267 | 9 | 90 | 3149 | Cros | Co | 2-59 months and 18-80 years | 3515 | SD Bioline Malaria Ag Pf | us-qPCR | High | Symptomatic |
| Landier et al., 2017 | Myanmar | 31 | 1 | 173 | 1237 | 71 | 4 | 133 | 1237 | Cros | Co | 27-48 years | 300 | SD Bioline Malaria Ag Pf | us-qPCR & Quansys ELISA | Low | Asymptomatic |
| Liu et al., 2019 | Myanmar | 28 | 45 | 37 | 1881 | 36 | 64 | 29 | 1862 | Cros | Co | 7 months to 90 years | 1991 | SD Bioline Malaria Ag Pf | nested-PCR | low | Asymptomatic&  Symptomatic |
| Manjurano et al., 2021 | Tanzania | 99 | 11 | 76 | 211 | 117 | 15 | 58 | 207 | Cros | Cl | >5 years | 397 | SD Bioline Malaria Ag Pf | 18S SSU rRNA nested-PCR | Moderate and high | Symptomatic |
| Owalla et al., 2020 | Uganda | 23 | 2 | 19 | 6 | 33 | 5 | 9 | 8 | Cros& Coh | Co | < 17 years | 50 | CareStart RDT | 18S rRNA us-qRTPCR | High | Asymptomatic |
| Rogier et al., 2020 | Haiti | 351 | 28 | 23 | 599 | 355 | 43 | 19 | 584 | NR | Co | NR | 1001 | SD Bioline Malaria Ag Pf | HRP2 immunoassay | low & High | NR |
| Unwin et al., 2020 | Indonesia | 36 | 5 | 107 | 122 | 31 | 2 | 127 | 110 | Ret | Cl | >15 years | 270 | CareStart RDT | 18S rDNA nested PCR | Medium | Asymptomatic |
| Vásquez et al., 2018 | Colombia | 27 | 0 | 8 | 702 | 30 | 4 | 5 | 698 | Ret | Cl | >15 years | 737 | SD Bioline Malaria Ag Pf | 18s rDNA nested-PCR | low | Asymptomatic and symptomatic |
| Vasquez et al., 2020 | Colombia | 21 | 0 | 18 | 819 | 25 | 1 | 14 | 813 | Cros | Cl | >15 years | 858 | SD Bioline Malaria Ag Pfst | 18s rDNA nested-PCR | low | Asymptomatic&  Symptomatic |
| Yeung et al., 2020 | Cambodia | 18 | 18 | 12 | 640 | 16 | 12 | 21 | 2677 | Cros | Co | <18 years | 2729 | SD Bioline Malaria Ag Pf | Cytochrome-b DNAnested PCR | low | Asymptomatic |

Abbreviations: TP, true positive; FP, false positive; FN, false negative; TN, true negative; NR, not reported; RDT, rapid diagnostic test; ELISA, enzyme-linked immunosorbent assay; DNA, deoxyribonucleic acid; RNA, Ribonucleic acid; PCR, polymerase chain reaction; Cros, cross-sectional; Coh, cohort; Ret, retrospective; Co, community based survey; Cl, clinical visit
