# Supplementary material for: Comparison of diagnostic performance between conventional and ultrasensitive rapid diagnostic tests for diagnosis of malaria: A systematic review and meta-analysis
Source: PLoS One. 2022 Feb 10;17(2):e0263770. doi: 10.1371/journal.pone.0263770 (PMC8830612; doi:10.1371/journal.pone.0263770)
Supplement: S1 File — (DOCX) [file pone.0263770.s003.docx]

**Study search strategies**

**PubMed (241)**

("infection plasmodium"[Title/Abstract] OR "plasmodium infections"[Title/Abstract] OR "malaria"[Title/Abstract]) AND ("ultra sensitive"[Title/Abstract] OR "ultrasensitive"[Title/Abstract] OR "highly sensitive"[Title/Abstract] OR "highly sensitive"[Title/Abstract] OR "high sensitive"[Title/Abstract] OR "high sensitive"[Title/Abstract]) AND ("diagnosis"[Title/Abstract] OR "detection"[Title/Abstract] OR "rapid diagnosis"[Title/Abstract] OR "rapid test"[Title/Abstract] OR "rapid diagnostic test"[Title/Abstract])

**Web of Sciences (785)**

TOPIC: (‘’Infection, Plasmodium’’ OR ‘’ Plasmodium Infections ‘’ OR ‘’ malaria ‘’) AND TOPIC: (‘’ ultra- sensitive’’ OR ''ultrasensitive'' OR ''highly-sensitive'' OR ''highly sensitive'' OR ''high-sensitive'' OR ''high sensitive'') AND TOPIC: (diagnosis OR detection OR ''rapid diagnosis'' OR ''rapid test'' OR ‘’rapid diagnostic test’’)

**Scopus (15)**

( TITLE-ABS-KEY ( ''infection, AND plasmodium'' OR '' AND plasmodium AND infections AND '' OR '' AND malaria AND '' ) ) AND ( TITLE-ABS-KEY ( '' AND ultra- AND sensitive'' OR ''ultrasensitive'' OR ''highly-sensitive'' OR ''highly AND sensitive'' OR ''high-sensitive'' OR ''high AND sensitive'' ) ) AND ( TITLE-ABS-KEY ( diagnosis OR detection OR ''rapid AND diagnosis'' OR ''rapid AND test'' OR ''rapid AND diagnostic AND test'' ) )

**Embase (424)**

No. Query Results

#4.          #1 AND #2 AND #3 424 31

#3.         ((('diagnosis'/exp OR diagnosis OR 464,835 31

       'detection'/exp OR detection OR 'rapid'/exp OR

          rapid) AND ('diagnosis'/exp OR diagnosis) OR

          'rapid'/exp OR rapid) AND ('test'/exp OR test) OR

        'rapid'/exp OR rapid) AND ('diagnostic'/exp OR

            diagnostic) AND ('test'/exp OR test)

#2.           ((ultra- AND sensitive OR ultrasensitive OR 315,777

          'highly sensitive' OR highly) AND sensitive OR

            'high sensitive' OR high) AND sensitive

#1.            (infection, AND ('plasmodium'/exp OR plasmodium) 134,933

               OR 'plasmodium'/exp OR plasmodium) AND

             ('infections'/exp OR infections) OR 'malaria'/exp

                 OR malaria

**ProQuest** (222)

ab(Infection, Plasmodium OR Plasmodium Infections OR malaria ) AND ab(ultra- sensitive OR ultrasensitive OR highly-sensitive OR highly sensitive OR high-sensitive OR high sensitive) AND ab(diagnosis OR detection OR rapid diagnosis OR rapid test OR rapid diagnostic test)
